# Self-reported adverse events associated with ∆^8^*-*Tetrahydrocannabinol (Delta-8-THC) Use

**DOI:** 10.1186/s42238-023-00191-y

**Published:** 2023-05-23

**Authors:** Eric C. Leas, Raquel M. Harati, Nora Satybaldiyeva, Nicolas E. Morales, Shelby L. Huffaker, Tomas Mejorado, Igor Grant

**Affiliations:** 1 Herbert Wertheim School of Public Health and Human Longevity Science, University of California, 9500 Gilman Drive, 0725, La Jolla, San Diego, CA 94304-1334 USA; 2 Qualcomm Institute, University of California, La Jolla, San Diego, CA USA; 3 California State University San Marcos, San Marcos, CA USA; 4 Center for Medicinal Cannabis Research, Department of Psychiatry, University of California, La Jolla, San Diego, CA USA

**Keywords:** Adverse Event, Cannabinoid, Delta-8-tetrahydrocannabinol, Reddit

## Abstract

**Background:**

There is an expanding unregulated market for a psychotropic compound called ∆^8^-Tetrahydrocannabinol (delta-8-THC) that is being derived from hemp, but a summary of adverse events related to delta-8-THC has not been publicly reported.

**Methods:**

This case series assessed adverse events reported by delta-8-THC users on the Reddit forum r/Delta8 and compared these to delta-8-THC AEs in the US Food and Drug Administration Adverse Event Reporting System (FAERS). Delta-8-THC and cannabis AEs reported in FAERS were also compared. The r/Delta8 forum was selected because it includes a large sample of 98,700 registered individuals who publicly discuss their experiences using delta-8-THC. All r/Delta8 posts were obtained from August 20, 2020, through September 25, 2022. A random sample of r/Delta8 posts was drawn (*n* = 10,000) and filtered for posts in which delta-8-THC users reported an adverse event (*n* = 335). FAERS reports that listed delta-8-THC (*N* = 326) or cannabis (*N* = 7076) as a suspect product active ingredient were obtained. Adverse events claimed to result from delta-8-THC use were coded using Medical Dictionary for Regulatory Activities to system organ class and preferred term categories.

**Results:**

The absolute number of delta-8-THC adverse event reports (*N* = 2184, 95% CI = 1949–2426) and serious adverse event reports (*N* = 437; 95% CI = 339–541) on r/Delta 8 were higher than the adverse event reports (*N* = 326) and serious adverse event reports (*N* = 289) to FAERS. Psychiatric disorders were the most frequently cited system organ class in r/Delta8 adverse event reports, mentioned in 41.2% (95% CI = 35.8%-46.3%) of reports, followed by respiratory, thoracic and mediastinal disorders (29.3%, 95% CI = 25.1%-34.0%) and nervous system disorders (23.3%, 95% CI = 18.5%-27.5%). Anxiety (16.4%, 95% CI = 12.8–20.6), Cough (15.5%, 95% CI = 11.9–20.0) and Paranoia (9.3%, 95% CI = 6.3–12.5) were the most frequently cited preferred terms in adverse event reports. The overall prevalence of AEs reported for cannabis and delta-8-THC on FAERS were also similar when analyzed by system organ class (Pearson’s r = 0.88).

**Conclusions:**

The findings of this case series suggest that most of the adverse events reported by delta-8-THC users are like those reported during acute cannabis intoxication. This finding suggests that health care professionals follow similar treatment and management protocols, and that jurisdictions should clarify whether delta-8-THC can be sold as a hemp product.

**Supplementary Information:**

The online version contains supplementary material available at 10.1186/s42238-023-00191-y.

## Background

Since 2020, the popularity of products containing the cannabis-derived chemical compound ∆^8^-Tetrahydrocannabinol (delta-8-THC) has exponentially increased in all 50 US states, particularly among US states that have restricted the use of the main psychotropic compound found in cannabis, ∆^9^-Tetrahydrocannabinol (delta-9-THC) (Leas et al. 2022). These increases are concomitant with marketing claims that delta-8-THC offers a “milder high” than delta-9-THC as well as claims by manufacturers that delta-8-THC has medicinal properties and can be sold legally as a “hemp” product and therefore without the restrictions applied to medical and recreational cannabis (Leas 2021; Harlow et al. 2022). Additionally, because they are unregulated most delta-8-THC products are not lab-tested like delta-9-THC products sold in state recreational cannabis systems and can be contaminated by other cannabinoids and heavy metals, (Meehan-Atrash and Rahman 2022) or mislabeled like other hemp-derived cannabinoid products.

After receiving 104 reports of adverse events (AEs) in patients who consumed delta-8-THC products between December 1, 2020 and February 28, 2022, the US Food and Drug Administration (FDA) issued a warning “for consumers to be aware that delta-8-THC products have not been evaluated or approved by the FDA for safe use in any context” (Office of the Commissioner 2022a; Spindle et al. 2022; Bonn-Miller et al. 2017). However, since this time, the only regulatory action taken by the FDA was to issue warning letters to a small number of companies that were adding delta-8-THC to food products (Office of the Commissioner 2022b). In the announcement of the warning letters, FDA Principal Deputy Commissioner Janet Woodcock stated that the agency “will continue to safeguard Americans’ health and safety by monitoring the marketplace and taking action when companies illegally sell products that pose a risk to public health.” Delayed regulatory action may in part result from a lack of available data documenting AEs related to delta-8-THC products.

To monitor the marketplace, the FDA uses a voluntary reporting system known as MedWatch, where health professionals, patients and consumers can report safety issues regarding products (Office of the Commissioner. 2022c). MedWatch reports are read by the FDA, coded, and then publicly released on the FDA Adverse Event Reporting System (FAERS) public dashboard (Center for Drug Evaluation and Research 2021). One previous report has described delta-8-THC cases listed in the FAERS database identifying an increase from 2019 to 2021 and that most AEs were respiratory (Simon et al. 2023). While MedWatch reports help the FDA identify safety signals and are one of the most efficient methods of capturing events associated with drug use, (Woodcock et al. 2011) the voluntary nature of the reporting system can lead to underreporting (Hazell and Shakir 2006).

To provide additional evidence for AEs related to delta-8-THC, we analyzed AEs posted on a social media website by self-identified delta-8-THC users as part of a case series study. Researchers are increasingly augmenting voluntary reporting systems like MedWatch with passive surveillance of social media, where consumers gather to discuss product use (Freifeld et al. 2014; Smith et al. 2018; Sarker et al. 2015). Large groups of delta-8-THC users are gathering on social media to openly discuss AEs that they have experienced while using delta-8-THC, including on Reddit, a social media website that receives 1.7 billion monthly site visits and is divided into topically-focused forums termed *subreddits* (Reddit.com web visitor traffic 2022). By reading these ongoing conversations, researchers can assess AEs that consumers have experienced, (Nobles et al. 2019; Leas et al. 2020) and by using a common medical language, can make comparisons between social media posts and FAERS (Freifeld et al. 2014; Smith et al. 2018). Herein, we sampled posts from the subreddit r/Delta8 in which registered individuals publicly discuss their experiences using delta-8-THC. We thematically analyzed the content of their posts to identify self-reported delta-8-THC users who experienced AEs, the severity of the AEs, and make comparisons to delta-8-THC AEs reported on FAERS. Finally, we make comparisons between delta-8-THC AEs and general cannabis-related AEs reported on FAERS.

## Methods

**Fig. 1:**
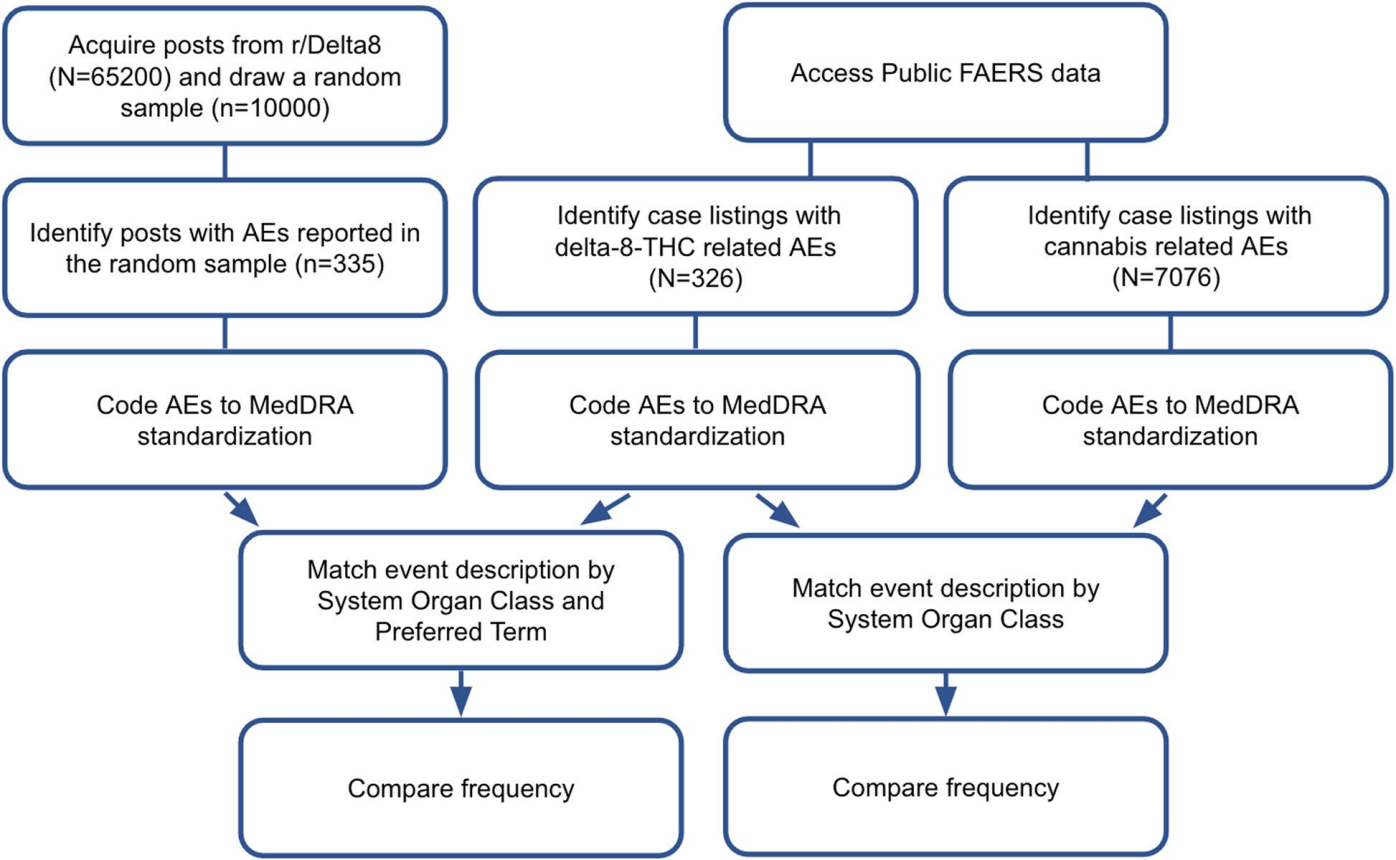
Data collection scheme for adverse events associated with delta-8-THC on Reddit and delta-8-THC and cannabis on FAERS. Abbreviations: AE = adverse event; r/Delta8 = a topically-focused Reddit forum known as a “subreddit” dedicated to discussions of delta-8-THC; FAERS = Food and Drug Administration Adverse Event Reporting System; MedDRA = Medical Dictionary for Regulatory Activities

Our approach was to first collect all English-language Reddit posts containing AEs in the primary subreddit for delta-8-THC, r/Delta8. Signs/symptoms in these posts were mapped to a standard regulatory dictionary. Then the aggregate frequency of identified AEs was compared with FAERS. Finally, cannabis and delta-8-THC AEs on FAERS were compared. A data collection schematic is shown in Fig. 1.

### Study samples

We obtained all r/Delta8 posts (*N* = 65,200) from April 14, 2020 (the inception of r/Delta8), through September 25, 2022 (the last day with publicly available data at the time of analysis). To identify AEs among delta-8-THC users, we randomly sampled 10,000 original posts, with a mean (SD) of 39 (84) words, to be further annotated. Comments were ignored because they are often nested in a stream of other comments and are typically briefer, thereby not including sufficient standalone details to be analyzed. To make comparisons to the FAERS voluntary reporting system, we obtained all case listing (*N* = 326) where delta-8-THC was a suspect product active ingredient that were provided in the FAERS public dashboard between August 2, 2011 (the date the FDA first received an AE case with delta-8-THC listed as a suspect product active ingredient and was indexed by FAERS) and September 25, 2022 (to match the r/Delta8 sample). We also obtained all case listings (*N* = 7076) where cannabis or cannabis flower was a suspect product active ingredient that were provided in the FAERS public dashboard between April 1, 1971 (the date the FDA first received an AE case with cannabis listed as a suspect product active ingredient and was indexed by FAERS) and September 25, 2022 (to match the delta-8-THC samples).

We checked for duplicate cases in FAERS by FDA case identification number, date of event, country of occurrence, age, and gender, but did not find any. The study was exempted from review and informed consent by the University of California, San Diego, Human Research Protections Program because the data were public and did not contain identifiable information (45 CFR §46). However, we edited direct quotations to avoid reverse identification (Ayers et al. 2018). This study followed the reporting guideline for case series (Kempen 2011).

### Adverse Event (AE) identification on Reddit

A post was considered to contain an AE if the user was describing their own personal use of delta-8-THC (vs., an anecdote about a friend) and they believed a negative health effect that was caused by their delta-8-THC use, which excluded any negative reviews they had about a product that were not related to health (e.g., they didn’t like the taste). Six annotators (E.C.L., R.M.H., N.S., N.E.M., S.L.H., and T.M.) coded an overlapping sample of 100 posts and unanimously agreed on the codes in all but 2 instances where 1 annotator disagreed (Fleiss’ K = 0.94). Because of the high agreement, each annotator reviewed their own independent sample of 1650 posts to identify posts containing AEs, resulting in *n* = 335 posts with a mean (SD) of 158 (183) words containing AEs that were retained for further analysis.

### Coding AEs on Reddit

We translated signs/symptoms reported in posts on r/Delta8 to a standardized medical language by using the Medical Dictionary for Regulatory Activities (MedDRA) English Language Version 25.1 (MedDRA n.d.). MedDRA is a dictionary of medical terminology developed under the auspices of the International Conference on Harmonization of Technical Requirements for Registration of Pharmaceuticals for Human Use. MedDRA is widely used by regulatory authorities for tracking AEs, including in FAERS (Center for Drug Evaluation and Research 2021). The MedDRA dictionary is organized by a 5-level hierarchy. “System Organ Class” (SOC) is the highest level of the terminology and represents an anatomical or physiological system, etiology, or purpose. Three levels below SOC are the “Preferred Terms” (PTs), which represents a single medical concept for a symptom, sign, or disease diagnosis. The “Lowest-Level Term” (LLT) is the lowest level of the terminology, reflecting how an observation is reported by a consumer in their own vernacular. All LLTs are linked to a single PT.

To translate r/Delta8 AEs to MedDRA, first four annotators (E.C.L., R.M.H., N.S., and N.E.M.) used open coding to develop a list of specific signs/symptoms relating to delta-8-THC use mentioned in posts, resulting in a list of *N* = 391 signs and symptoms (Charmaz 2006). Then three annotators (E.C.L., R.M.H. and N.S.) assigned all signs/symptoms to a MedDRA LLTs. There was no restriction on the number of signs/symptoms or LLTs a post could contain. For example, the post “I vaped delta-8-THC and then started coughing and got anxious,” would be mapped to the LLTs “Coughing” and “Anxiety” by identifying “coughing” and “anxious” as signs/symptoms. Annotators had moderate agreement on LLT selections (Cohen’s K = 0.61), with difference primarily resulting from lexical variations allowed in MedDRA (e.g., MedDRA has LLTs for “cough” and “coughing”). Agreement was higher the PT (Cohen’s K = 0.76) and SOC (Cohen’s K = 0.86), therefore analyses were performed at these levels.

### AE severity rating on Reddit

Four annotators (E.C.L., R.M.H., N.S., and N.E.M.) labeled the severity of the AEs using the criteria provided in the MedWatch reports: hospitalization; birth-defect or congenital abnormality; required help to prevent permanent harm, disability, or health problem; life-threatening; another serious/important medical incident. MedWatch also includes a category for death which was excluded per our requirement that posts be self-authored by delta-8-THC users. Posts that were labeled with any of the 5 categories were considered “serious” AEs. Agreement on which posts were serious AEs was high (Prevalence-Bias-Adjusted Fleiss K = 0.77) (Byrt et al. 1993).

### AE identification on FAERS

Using the data from FAERS, we considered all reactions presented in case listing as well as the date the FDA initially received information regarding the case, whether the case was considered serious and the outcomes associated with the case (e.g., hospitalization).

### Statistical analysis

We used the sample of r/Delta8 posts and bootstrapping to estimate the proportion and frequency of AEs and serious AEs in the full archive and make comparisons between these using ratios. Among posts that mentioned AEs, we estimate the proportion within each primary SOC and PT. Among the sample of reports associated with delta-8-THC, we also assess the correlation in the prevalence of SOCs between r/Delta8 sample and FAERS using the Pearson’s correlation coefficient. We also report the mean number of PTs associated with each AE in FAERS and r/Delta8, make comparison between these averages using a ratio and report the number of PTs found in r/Delta8 that were not listed in FAERS. Finally, we assess the correlation in the prevalence of SOCs between delta-8-THC and cannabis on FAERS using the Pearson’s correlation coefficient. All analyses were preformed using R, version 4.1.1 (R Project for Statistical Computing).

## Results

**Fig. 2:**
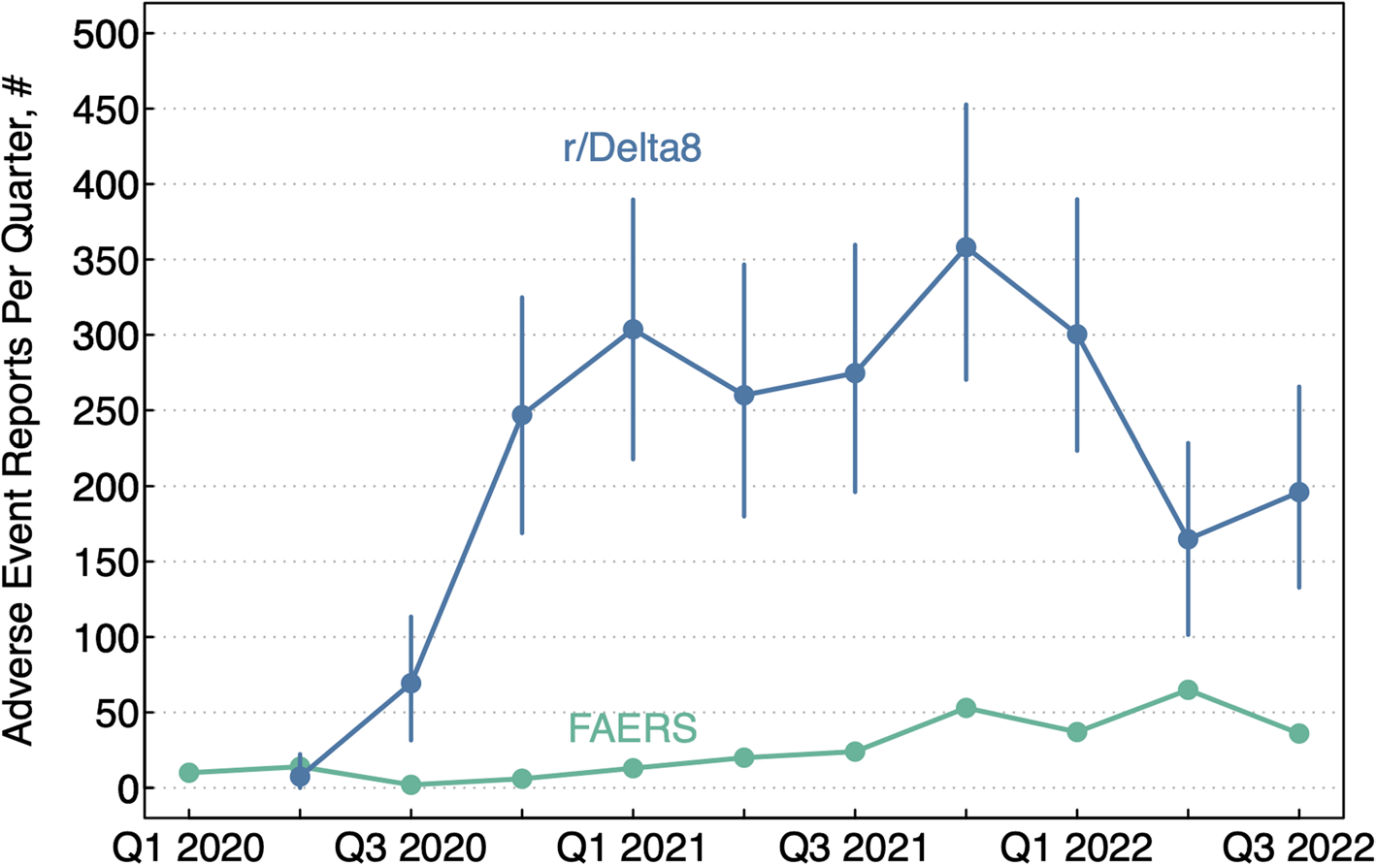
The number of adverse events associated with delta-8-THC reported on FAERS and estimated number of adverse events associated with delta-8-THC reported on the Reddit delta-8-THC forum r/Delta8 by quarter. Abbreviations: r/Delta8 = a topically-focused Reddit forum known as a “subreddit” dedicated to discussions of delta-8-THC; FAERS = Food and Drug Administration Adverse Event Reporting System

Using the random sample of r/Delta8 posts, we estimated that 3.4% (95% CI = 3.0%-3.7%) of all r/Delta8 posts were AE reports and 0.7% (95% CI = 0.5%-0.9%) were serious AE reports. The proportion of AE reports on r/Delta8 that were considered serious (20.0%, 95% CI = 15.8%-24.2%) was lower than FAERS (88.7%), but the estimated absolute number of AE reports (*N* = 2184, 95% CI = 1949–2426) and serious AE reports (*N* = 437, 95% CI = 339–541) on r/Delta 8 was higher than the number of AE reports (*N* = 326) and serious AE reports (*N* = 289) on FAERS. Cumulatively, 56 delta-8-THC AE reports were recorded on FAERS prior to the inception of r/Delta8 in Q2 2020 (Fig. 2). In the first quarter after the inception of r/Delta8, the number of AE reports on r/Delta8 (*N* = 7, 95% CI = 0–22) and FAERS (*N* = 14) was not statistically different, but by Q3 2020 the estimated number of AE reports on r/Delta8 (N = 69, 95% CI = 32–113) was significantly higher than the number of AE reports on FAERS (*N* = 2) and remained significantly higher in every quarter until the end of the study period (Q3 2022). For instance, in the last quarter of the study period, the number of AE reports on r/Delta8 (*N* = 196, 95% CI = 133–266) was 5.4 times as high the number of AE reports on FAERS (*N* = 36).

Psychiatric disorders were the most common category of AEs reported on r/Delta8, mentioned in 41.2% (95% CI = 35.8%-46.3%) of all reports (Table 1), including “I got so high on Delta8 that I thought I was going to die. I called 911 and on the way to the ER they told me I might be having a panic attack." The second most cited subcategory was respiratory, thoracic and mediastinal disorders (29.3%, 95% CI = 25.1%-34.0%), followed by nervous system disorders (23.3%, 95% CI = 18.5%-27.5%), general disorders and administration site conditions (22.1%, 95% CI = 17.8%-26.6%), gastrointestinal disorders (17.0%, 95% CI = 12.8%-21.2%), injury, poisoning and procedural complications (8.7%, 95% CI = 5.7%-11.9%), investigations (e.g., blood pressure elevated) (7.5%, 95% CI = 4.8%-10.1%), and eye disorders (6.0%, 95% CI = 3.3%-8.7%). All other categories of SOCs were cited < 5% of posts on r/Delta8.

**Table 1:** Adverse events associated with delta-8-THC reported by delta-8-THC users on Reddit forum r/Delta8 by MedDRA System Organ Class

MedDRA System Organ Class	MedDRA Preferred Terms	Relevant Example Text^b^	Category Prevalence
Psychiatric disorders	Abnormal dreams, Agitation, Anxiety, Apathy, Autoscopy^a^, Bruxism^a^, Delusion, Dependence^a^, Depersonalisation/derealisation disorder, Depressed mood^a^, Depression, Derealisation^a^, Disorientation, Dissociation, Dysphemia^a^, Emotional poverty^a^, Fear, Hallucination, Hallucination visual, Hallucinations mixed, Illusion, Insomnia, Irritability, Laziness^a^, Libido increased^a^, Mood swings, Negative thoughts, Nervousness, Nightmare^a^, Panic attack, Panic reaction, Paranoia, Poor quality sleep, Post-traumatic stress disorder, Sleep attacks^a^, Tachyphrenia, Time perception altered	"I'm no lightweight, but I got so high on Delta8 that I thought I was going to die. I called 911 and on the way to the ER they told me I might be having a panic attack." (PTs: Anxiety, Panic Attack)	41.2% (95% CI: 35.8–46.3)
Respiratory, thoracic and mediastinal disorders	Asthma^a^, Choking sensation, Cough, Dry throat^a^, Dyspnoea, Epistaxis, Haemoptysis^a^, Nasal discomfort^a^, Oropharyngeal discomfort^a^, Oropharyngeal pain, Painful respiration^a^, Pharyngeal swelling^a^, Pneumonitis^a^, Productive cough^a^, Pulmonary pain^a^, Respiratory tract irritation, Rhinalgia^a^, Rhinorrhoea^a^, Sinus pain^a^, Sneezing^a^, Sputum discoloured^a^, Throat irritation^a^, Throat tightness	“It began to hurt to breath, my lungs were hurting, and my throat was tight. I went to the ER for the pain, it was that bad.” (PTs: Painful respiration, Pulmonary pain, Throat tightness)	29.3% (95% CI: 25.1–34.0)
Nervous system disorders	Ageusia^a^, Altered state of consciousness, Amnesia, Balance disorder, Burning sensation, Disturbance in attention, Dizziness, Dysarthria, Formication, Head discomfort^a^, Headache, Hepatic encephalopathy^a^, Hypoaesthesia, Loss of consciousness, Neuralgia^a^, Neurological symptom^a^, Non-24-h sleep–wake disorder^a^, Paraesthesia, Paralysis, Presyncope, Psychomotor hyperactivity, Sedation, Seizure, Sensory disturbance, Somnolence, Tremor	“The high hit me all at once like a semi-truck. I was paralyzed and my legs were shaking uncontrollably‚ then I had a full on seizure which was later confirmed by a doctor.” (PTs: Paralysis, Tremor, Seizure)	23.3% (95% CI: 18.5–27.5)
General disorders and administration site conditions	Axillary pain^a^, Chest discomfort, Chest pain, Chills, Drug withdrawal syndrome, Energy increased^a^, Fatigue, Feeling abnormal, Feeling hot, Feeling jittery, Hangover^a^, Hunger, Ill-defined disorder^a^, Pain, Pyrexia, Secretion discharge^a^, Thirst, Thirst decreased^a^, Unevaluable event, Withdrawal syndrome	"I tried to quit d8 cold turkey but beware of these withdrawals. I relapsed after nine days because of how bad it was." (PTs: Drug withdrawal syndrome)	22.1% (95% CI: 17.9–26.6)
Gastrointestinal disorders	Abdominal discomfort, Abdominal distension^a^, Abdominal pain, Abdominal pain upper, Constipation, Diarrhoea, Dry mouth, Flatulence^a^, Gastrooesophageal reflux disease, Intra-abdominal fluid collection^a^, Nausea, Odynophagia^a^, Salivary hypersecretion^a^, Swollen tongue, Tongue discomfort^a^, Tooth deposit^a^, Vomiting	“I’ve been having some bad stomach pain, dry mouth, and diarrhoea lately the last few times I've used d8." (PTs: Abdominal pain, dry mouth, diarrhoea)	17.0% (95% CI: 12.8–21.2)
Injury, poisoning and procedural complications	Accidental overdose	“How is this stuff legal? I'm so high right now and I've been high for the last 3 days since I first tried the edibles. I accidentally took way too much.” (PT: Accidental overdose)	8.7% (95% CI: 5.7–11.9)
Investigations	Ammonia increased^a^, Blood pressure increased, Heart rate increased, Weight increased^a^	"My watch told me my heart rate was at 162 while I was sitting down! My heart felt as if it was beating out of my chest." (PT: Heart rate increased)	7.5% (95% CI: 4.8–10.1)
Eye disorders	Asthenopia^a^, Blepharospasm^a^, Dry eye^a^, Eye irritation, Eye pain^a^, Eyelid ptosis^a^, Metamorphopsia^a^, Ocular hyperaemia, Photophobia^a^, Photopsia^a^, Vision blurred, Visual impairment	“It was awful, my eyes began to hurt bad, the pain felt someone was pouring acid in my eyes. Then they felt dry for days.“ (PTs: Eye pain, Dry Eye)	6.0% (95% CI: 3.3–8.7)
Metabolism and nutrition disorders	Abnormal loss of weight^a^, Appetite disorder^a^, Decreased appetite, Dehydration, Food craving^a^, Increased appetite^a^	"When I'm on delta-8, I go days without food and feel completely normal as long as I'm high still." (PT: Decreased Appettite)	4.5% (95% CI: 2.7–6.6)
Skin and subcutaneous tissue disorders	Acne^a^, Cold sweat, Hyperhidrosis, Night sweats, Piloerection^a^, Rash, Rash erythematous^a^, Urticaria	"I was getting these cold sweats and goose bumps all over my body." (PTs: Cold sweat, Piloerection)	3.3% (95% CI: 1.5–5.1)
Musculoskeletal and connective tissue disorders	Muscle spasms, Muscle twitching, Pain in extremity^a^	"Both of my entire legs cramped up multiple times during my trip on the edibles, it was extremely painful." (PTs: Muscle spasms, Pain in extremity)	2.4% (95% CI: 0.9–4.2)
Ear and labyrinth disorders	Hyperacusis^a^, Tinnitus, Vertigo	"I couldn't shake the vertigo, the room was spinning so much. It was not enjoyable at all." (PT: Vertigo)	2.1% (95% CI: 0.9–3.9)
Vascular disorders	Hypertension, Shock, Vascular pain^a^	"I had a really bad reaction to delta 8, I've been feeling pain in my veins and arteries for the past week." (PT: Vascular pain)	1.2% (95% CI: 0.3–2.7)
Hepatobiliary disorders	Hepatomegaly^a^, Perihepatic discomfort^a^	“The ER did CT scans and they found was an enlarged liver which the Dr. said could be due to the d8 use.” (PT: Hepatomegaly)	0.6% (95% CI: 0.0–1.5)
Cardiac disorders	Palpitations	"Had a gummy last night and woke up with heart palipitations. Waiting to hear back from my Doctor on what to do." (PT: Palpitations)	0.6% (95% CI: 0.0–1.5)
Immune system disorders	Hypersensitivity	"Right after vaping it felt like I was having an allergic reaction." (PT: Hypersensitivity)	0.3% (95% CI: 0.0–0.9)
Infections and infestations	Sinusitis	"Vaped 2 weeks ago and have had a sinus infection and sinus pain since then.” (PT: Sinustis)	0.3% (95% CI: 0.0–0.9)
Renal and urinary disorders	Urinary retention^a^	"I felt like I needed to urinate but when I tried to go, I couldn't go at all." (PT: Urinary Retention)	0.3% (95% CI: 0.0–0.9)

^a^Indicates a MedDRA preferred term that was not listed in a FAERS case

^b^Each post was edited to omit content that might make the author identifiable

*Abbreviations*: *ER* Emergency Room, *CT scan* Computerized tomography scan, *FAERS* Food and Drug Administration Adverse Event Reporting System, *MedDRA* Medical Dictionary for Regulatory Activities, *PT* MedDRA Preferred term

In Table 2 we list the PTs mentioned in AE reports on r/Delta8. The PTs cited in at least 5% of AE reports on r/Delta8 included Anxiety (16.4%, 95% CI = 12.8–20.6), Cough (15.5%, 95% CI = 11.9–20.0), Paranoia (9.3%, 95% CI = 6.3–12.5), Accidental Overdose (8.7%, 95% CI = 5.7–11.6), Heart Rate Increased (6.9%, 95% CI = 4.5–9.6), Panic attack (6.9%, 95% CI = 4.5–9.9), Dyspnoea (5.7%, 95% CI = 3.3–8.1), Headache (5.4%, 95% CI = 3.0–7.8), Nausea (5.4%, 95% CI = 3.0–8.1) and Vomiting (5.4%, 95% CI = 3.3–8.1).

**Table 2:** Adverse events associated with delta-8-THC reported by delta-8-THC users on Reddit delta-8-THC forum r/Delta8 by MedDRA Preferred Term

MedDRA Preferred Terms	Subcategory Prevalence
Anxiety	16.4% (95% CI: 12.8–20.6)
Cough	15.5% (95% CI: 11.6–19.4)
Paranoia	9.3% (95% CI: 6.3–12.2)
Accidental overdose	8.7% (95% CI: 5.7–11.9)
Heart rate increased	6.9% (95% CI: 4.5–9.9)
Panic attack	6.9% (95% CI: 4.2–9.6)
Dyspnoea	5.7% (95% CI: 3.3–8.4)
Headache	5.4% (95% CI: 3.0–7.8)
Nausea	5.4% (95% CI: 3.0–7.8)
Vomiting	5.4% (95% CI: 3.0–7.8)
Dizziness	5.1% (95% CI: 3.0–7.5)
Feeling abnormal	4.8% (95% CI: 2.7–7.2)
Throat irritation^a^	4.2% (95% CI: 2.1–6.6)
Pulmonary pain^a^	3.9% (95% CI: 2.1–6.0)
Chest pain	3.6% (95% CI: 1.8–5.7)
Amnesia	3.3% (95% CI: 1.5–5.4)
Chest discomfort	3.0% (95% CI: 1.5–5.1)
Fatigue	3.0% (95% CI: 1.2–4.8)
Tremor	3.0% (95% CI: 1.2–4.8)
Depression	2.7% (95% CI: 0.9–4.5)
Panic reaction	2.7% (95% CI: 1.2–4.5)
Withdrawal syndrome	2.7% (95% CI: 1.2–4.5)
Disturbance in attention	2.4% (95% CI: 0.9–4.2)
Unevaluable event	2.4% (95% CI: 0.9–4.2)
Abnormal dreams	2.1% (95% CI: 0.6–3.6)
Insomnia	2.1% (95% CI: 0.6–3.9)
Decreased appetite	1.8% (95% CI: 0.6–3.3)
Hypoaesthesia	1.8% (95% CI: 0.6–3.3)
Chills	1.8% (95% CI: 0.6–3.3)
Depersonalisation/derealisation disorder	1.8% (95% CI: 0.6–3.3)
Somnolence	1.8% (95% CI: 0.6–3.3)
Dry mouth	1.8% (95% CI: 0.6–3.3)
Metamorphopsia^a^	1.8% (95% CI: 0.6–3.6)
Dependence^a^	1.5% (95% CI: 0.3–3.0)
Increased appetite^a^	1.5% (95% CI: 0.3–3.0)
Diarrhoea	1.5% (95% CI: 0.3–3.0)
Head discomfort^a^	1.5% (95% CI: 0.3–3.0)
Respiratory tract irritation	1.5% (95% CI: 0.3–3.0)
Productive cough^a^	1.5% (95% CI: 0.3–3.0)
Oropharyngeal pain	1.5% (95% CI: 0.3–2.7)
Abdominal pain upper	1.5% (95% CI: 0.3–2.7)
Abdominal discomfort	1.5% (95% CI: 0.3–3.0)
Hyperhidrosis	1.5% (95% CI: 0.3–3.0)
Muscle twitching	1.5% (95% CI: 0.3–3.0)
Time perception altered	1.5% (95% CI: 0.3–3.0)
Vision blurred	1.2% (95% CI: 0.3–2.4)
Drug withdrawal syndrome	1.2% (95% CI: 0.3–2.7)
Dysarthria	1.2% (95% CI: 0.3–2.4)
Hallucination, visual	1.2% (95% CI: 0.3–2.4)
Autoscopy^a^	1.2% (95% CI: 0.3–2.7)
Abdominal pain	0.9% (95% CI: 0.0–2.1)
Hallucinations, mixed	0.9% (95% CI: 0.0–2.1)
Loss of consciousness	0.9% (95% CI: 0.0–1.8)
Hypertension	0.9% (95% CI: 0.0–2.1)
Ocular hyperaemia	0.9% (95% CI: 0.0–2.1)
Seizure	0.9% (95% CI: 0.0–2.1)
Disorientation	0.9% (95% CI: 0.0–1.8)
Dissociation	0.9% (95% CI: 0.0–1.8)
Irritability	0.9% (95% CI: 0.0–2.1)
Muscle spasms	0.9% (95% CI: 0.0–2.1)
Paraesthesia	0.9% (95% CI: 0.0–2.1)
Tinnitus	0.9% (95% CI: 0.0–2.1)
Sedation	0.9% (95% CI: 0.0–2.1)
Sneezing^a^	0.9% (95% CI: 0.0–2.1)
Vertigo	0.9% (95% CI: 0.0–2.1)
Pain	0.6% (95% CI: 0.0–1.5)
Agitation	0.6% (95% CI: 0.0–1.5)
Choking sensation	0.6% (95% CI: 0.0–1.5)
Delusion	0.6% (95% CI: 0.0–1.5)
Depressed mood^a^	0.6% (95% CI: 0.0–1.5)
Blood pressure increased	0.6% (95% CI: 0.0–1.5)
Thirst	0.6% (95% CI: 0.0–1.5)
Flatulence^a^	0.6% (95% CI: 0.0–1.5)
Hunger	0.6% (95% CI: 0.0–1.5)
Nervousness	0.6% (95% CI: 0.0–1.5)
Palpitations	0.6% (95% CI: 0.0–1.5)
Dysphemia^a^	0.6% (95% CI: 0.0–1.5)
Bruxism^a^	0.6% (95% CI: 0.0–1.5)
Ill-defined disorder^a^	0.6% (95% CI: 0.0–1.5)
Blepharospasm^a^	0.6% (95% CI: 0.0–1.5)
Food craving^a^	0.6% (95% CI: 0.0–1.5)
Presyncope	0.6% (95% CI: 0.0–1.5)
Tachyphrenia	0.6% (95% CI: 0.0–1.5)
Abnormal loss of weight^a^	0.6% (95% CI: 0.0–1.5)
Emotional poverty^a^	0.6% (95% CI: 0.0–1.5)
Balance disorder	0.6% (95% CI: 0.0–1.5)
Sensory disturbance	0.3% (95% CI: 0.0–0.9)
Acne^a^	0.3% (95% CI: 0.0–0.9)
Hypersensitivity	0.3% (95% CI: 0.0–0.9)
Altered state of consciousness	0.3% (95% CI: 0.0–0.9)
Ammonia increased^a^	0.3% (95% CI: 0.0–0.9)
Abdominal distension^a^	0.3% (95% CI: 0.0–1.2)
Eye irritation	0.3% (95% CI: 0.0–0.9)
Tongue discomfort^a^	0.3% (95% CI: 0.0–0.9)
Cold sweat	0.3% (95% CI: 0.0–0.9)
Constipation	0.3% (95% CI: 0.0–0.9)
Haemoptysis^a^	0.3% (95% CI: 0.0–0.9)
Formication	0.3% (95% CI: 0.0–0.9)
Dehydration	0.3% (95% CI: 0.0–0.9)
Non-24-h sleep–wake disorder^a^	0.3% (95% CI: 0.0–0.9)
Dry eye^a^	0.3% (95% CI: 0.0–0.9)
Dry throat^a^	0.3% (95% CI: 0.0–0.9)
Asthma^a^	0.3% (95% CI: 0.0–0.9)
Burning sensation	0.3% (95% CI: 0.0–0.9)
Eye pain^a^	0.3% (95% CI: 0.0–0.9)
Asthenopia^a^	0.3% (95% CI: 0.0–0.9)
Fear	0.3% (95% CI: 0.0–0.9)
Feeling jittery	0.3% (95% CI: 0.0–0.9)
Feeling hot	0.3% (95% CI: 0.0–0.9)
Pyrexia	0.3% (95% CI: 0.0–0.9)
Photopsia^a^	0.3% (95% CI: 0.0–1.2)
Hallucination	0.3% (95% CI: 0.0–0.9)
Hangover^a^	0.3% (95% CI: 0.0–0.9)
Hepatic encephalopathy^a^	0.3% (95% CI: 0.0–0.9)
Urticaria	0.3% (95% CI: 0.0–0.9)
Psychomotor hyperactivity	0.3% (95% CI: 0.0–0.9)
Salivary hypersecretion^a^	0.3% (95% CI: 0.0–0.9)
Apathy	0.3% (95% CI: 0.0–0.9)
Hepatomegaly^a^	0.3% (95% CI: 0.0–0.9)
Ageusia^a^	0.3% (95% CI: 0.0–0.9)
Mood swings	0.3% (95% CI: 0.0–0.9)
Nasal discomfort^a^	0.3% (95% CI: 0.0–0.9)
Rhinalgia^a^	0.3% (95% CI: 0.0–0.9)
Neuralgia^a^	0.3% (95% CI: 0.0–1.2)
Night sweats	0.3% (95% CI: 0.0–0.9)
Nightmare^a^	0.3% (95% CI: 0.0–0.9)
Epistaxis	0.3% (95% CI: 0.0–0.9)
Pain in extremity^a^	0.3% (95% CI: 0.0–0.9)
Painful respiration^a^	0.3% (95% CI: 0.0–0.9)
Illusion	0.3% (95% CI: 0.0–0.9)
Post-traumatic stress disorder	0.3% (95% CI: 0.0–0.9)
Eyelid ptosis^a^	0.3% (95% CI: 0.0–1.2)
Rhinorrhoea^a^	0.3% (95% CI: 0.0–0.9)
Libido increased^a^	0.3% (95% CI: 0.0–0.9)
Shock	0.3% (95% CI: 0.0–0.9)
Sinusitis	0.3% (95% CI: 0.0–0.9)
Sinus pain^a^	0.3% (95% CI: 0.0–0.9)
Sleep attacks^a^	0.3% (95% CI: 0.0–0.9)
Poor quality sleep	0.3% (95% CI: 0.0–0.9)
Odynophagia^a^	0.3% (95% CI: 0.0–0.9)
Swollen tongue	0.3% (95% CI: 0.0–0.9)
Pharyngeal swelling^a^	0.3% (95% CI: 0.0–0.9)
Throat tightness	0.3% (95% CI: 0.0–0.9)
Vascular pain^a^	0.3% (95% CI: 0.0–0.9)
Visual impairment	0.3% (95% CI: 0.0–0.9)
Weight increased^a^	0.3% (95% CI: 0.0–0.9)
Piloerection^a^	0.3% (95% CI: 0.0–0.9)
Energy increased^a^	0.3% (95% CI: 0.0–0.9)
Thirst decreased^a^	0.3% (95% CI: 0.0–0.9)
Laziness^a^	0.3% (95% CI: 0.0–0.9)
Perihepatic discomfort^a^	0.3% (95% CI: 0.0–0.9)
Derealisation^a^	0.3% (95% CI: 0.0–0.9)
Negative thoughts	0.3% (95% CI: 0.0–0.9)
Rash	0.3% (95% CI: 0.0–1.2)
Tooth deposit^a^	0.3% (95% CI: 0.0–0.9)
Neurological symptom^a^	0.3% (95% CI: 0.0–0.9)
Photophobia^a^	0.3% (95% CI: 0.0–0.9)
Intra-abdominal fluid collection^a^	0.3% (95% CI: 0.0–0.9)
Gastrooesophageal reflux disease	0.3% (95% CI: 0.0–1.2)
Paralysis	0.3% (95% CI: 0.0–0.9)
Hyperacusis^a^	0.3% (95% CI: 0.0–1.2)
Secretion discharge^a^	0.3% (95% CI: 0.0–0.9)
Appetite disorder^a^	0.3% (95% CI: 0.0–0.9)
Sputum discoloured^a^	0.3% (95% CI: 0.0–0.9)
Urinary retention^a^	0.3% (95% CI: 0.0–0.9)
Pneumonitis^a^	0.3% (95% CI: 0.0–0.9)
Oropharyngeal discomfort^a^	0.3% (95% CI: 0.0–0.9)
Rash erythematous^a^	0.3% (95% CI: 0.0–1.2)
Axillary pain^a^	0.3% (95% CI: 0.0–0.9)

^a^Indicates a MedDRA preferred term that was not listed in a FAERS case

*Abbreviations*: *FAERS* Food and Drug Administration Adverse Event Reporting System, *MedDRA* Medical Dictionary for Regulatory Activities

**Fig. 3:**
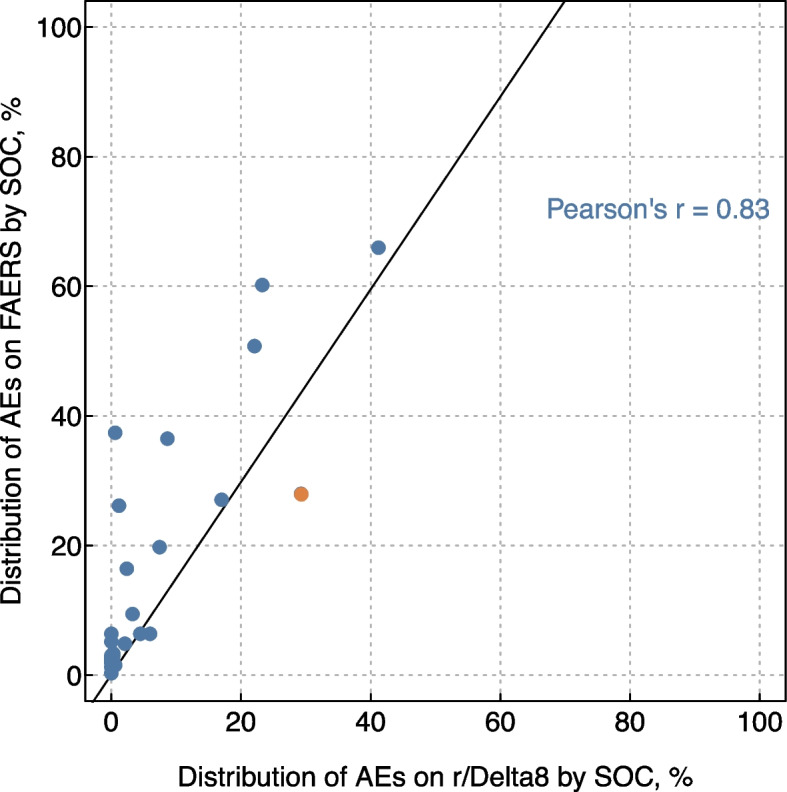
The overall prevalence of adverse events associated with delta-8-THC reported on r/Delta8 and FAERS were similar when analyzed by MedDRA System Organ Class. Note: each dot is the number of all AE reports associated with delta-8-THC labeled with each MedDRA SOC in FAERS (y-axis) or r/Delta8 (x-axis) divided by the total number of AE reports associated with delta-8-THC on FAERS (y-axis) or r/Delta8 (x-axis). For instance, 29.3% of all delta-8-THC AEs reports in the r/Delta8 sample and 27.9% of all delta-8-THC AE reports in FAERS were categorized as respiratory, thoracic, and mediastinal disorders (highlighted in orange). The line represents the linear correlation in the prevalence of each SOCs between the two samples and is summarized with a Pearson’s r statistic. Abbreviations: AE = adverse event; r/Delta8 = a topically-focused Reddit forum known as a “subreddit” dedicated to discussions of delta-8-THC; FAERS = Food and Drug Administration Adverse Event Reporting System; MedDRA = Medical Dictionary for Regulatory Activities; SOC = MedDRA System Organ Class

The overall prevalence of AEs reported on r/Delta8 and FAERS were similar when analyzed by SOC, with a strong correlation (Pearson’s *r* = 0.83) in prevalence across category of SOC (Fig. 3). For instance, 29.3% of all delta-8-THC AEs reports in the r/Delta8 sample and 27.9% of all delta-8-THC AE reports in FAERS were categorized as respiratory, thoracic, and mediastinal disorders. Although the FAERS AE reports received 2.4 times as many PTs on average as the r/Delta8 reports (mean PTs FAERS reports = 5.3, 95% = 4.8–6.0) vs. mean PTs r/Delta8 reports = 2.2, 95% CI = 2.1–2.4), 69 of the 169 unique PTs identified on r/Delta8 were not found among PTs listed on FAERS reports (denoted with asterisks in Table 1). Among those not listed in FAERS, 29 were linked to reports that were considered serious AEs. In the Supplementary Table e1, we list all 67 serious AEs identified in the sample according to the implicated SOCs and PTs and the outcome of the AE.

**Fig. 4:**
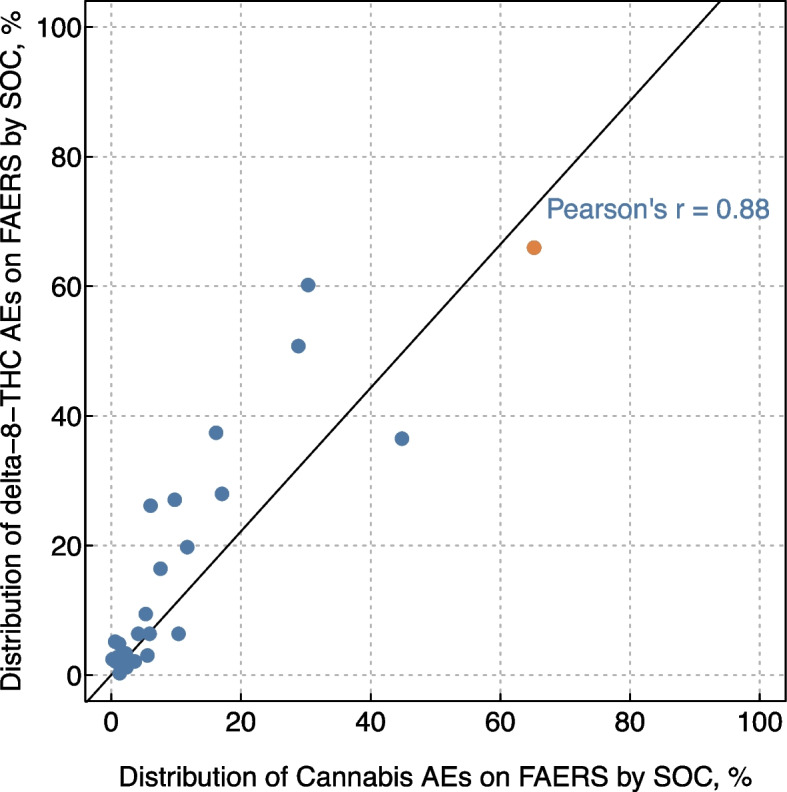
The overall prevalence of adverse events associated with delta-8-THC and cannabis reported on FAERS were similar when analyzed by MedDRA System Organ Class. Note: each dot is the number of all AE reports associated with delta-8-THC (y-axis) or cannabis (x-axis) in FAERS labeled with each MedDRA SOC divided by the total number of AE reports associated with delta-8-THC or cannabis respectively. For instance, psychiatric disorders were the most prevalent AE reported for both delta-8-THC and cannabis cases, being reported by 66.0% of all delta-8-THC AEs reports and 65.2% of all cannabis AE reports in FAERS (highlighted in orange). The line represents the linear correlation in the prevalence of each SOCs between the two samples and is summarized with a Pearson’s r statistic. Abbreviations: AE = adverse event; r/Delta8 = a topically-focused Reddit forum known as a “subreddit” dedicated to discussions of delta-8-THC; FAERS = Food and Drug Administration Adverse Event Reporting System; MedDRA = Medical Dictionary for Regulatory Activities; SOC = MedDRA System Organ Class

The overall prevalence of AEs reported for cannabis and delta-8-THC on FAERS were also similar when analyzed by SOC, with a strong correlation (Pearson’s *r* = 0.88) in prevalence across category of SOC (Fig. 4). For instance, psychiatric disorders were the most prevalent AE reported for both delta-8-THC and cannabis cases, being reported by 66.0% of all delta-8-THC AEs reports and 65.2% of all cannabis AE reports in FAERS.

## Discussion

The public has increasingly taken to the social media platform Reddit to report AEs they believe resulted from using delta-8-THC, with an increase in the number of AE reports mirroring the increase and trajectory of popular interest in delta-8-THC (Leas et al. 2022). Typically, the AEs reported on Reddit were less serious than those reported to FAERS, but given the volume of posts, the absolute number of serious AEs reported was estimated to be higher. When comparing the distribution of the broad categories of AEs, posts on the delta-8-THC forum on Reddit and FAERS reports were similar. However, several new subcategories were found on the forum that had not been previously captured on FAERS. These results provide insights that could inform clinical practice and regulation of delta-8-THC.

Many of the AEs reported for delta-8-THC on r/Delta8 and FAERS were like those that can occur during acute intoxication of delta-9-THC-containing cannabis. For example, the top-level categories of health conditions were very similar between the AEs listing cannabis on FAERS and the AEs listing delta-8-THC on FAERS, with psychiatric conditions being the most prevalent category for each. Similarly, the 3 most common PTs identified on r/Delta8–Anxiety, Cough, and Paranoia–were also the most commonly reported symptoms in a recent survey of acute adverse reactions to delta-9-THC-containing cannabis (LaFrance et al. 2020). For this reason, effective treatment and management of delta-8-THC cases may be similar to that of acute intoxication of delta-9-THC which typically does not require diagnostic testing and consists of symptom management (Turner et al. 2022). In cases involving psychiatric disorders–the most prevalent SOC cited among delta-8-THC AE reports–the typical period of cannabis detoxication is 24 h but can be longer if symptoms persist or vital signs are unstable (Noble et al. 2019). Among chronic cannabis users, withdrawal symptoms, which were reported among several AEs on r/Delta8, can occur between 24–48 h after cessation and generally peak at days 2–6. These symptoms can require their own symptom management (Connor et al. 2022). Because delta-8-THC appears to affect many organ systems, a multi-disciplinary team including internists, psychiatrists, and occasionally cardiologists should assist with managing cases.

Several regulatory challenges add context to our findings. Delta-8-THC products are sold without many of the safeguards that have been instituted by state-run recreational cannabis programs (Leas 2021). This includes a lack of testing requirements for potency, consistency, and presence of contaminants, which could explain why the fourth most mentioned PT among the AEs reported on r/Delta8 was “accidental overdose.” There is evidence that adulterated products may be present in the supply chain (Meehan-Atrash and Rahman 2022) and the result of this contamination could change the clinical profile and treatment of delta-8-THC cases. For example, illicit cannabis vaping products containing the cutting agent vitamin E acetate were determined to be the primary cause of the 2019 outbreak of lung injury known as EVALI that led to 2668 hospitalizations and 68 deaths (CDC’s Office on Smoking and Health 2020). The FAERS delta-8-THC data indexed one case that included EVALI as a PT, suggesting delta-8-THC products could also be implicated in this outbreak. Without product standards for delta-8-THC manufacturing and synthesis, other contaminants could lead to new outbreaks. The lack of track-and-trace system infrastructure for sourcing product batches and removing contaminated batches from the supply chain can delay regulatory responses as demonstrated during EVALI. These dangers demand regulatory actions governing when, where, and how delta-8-THC is sold, consistent with the actions taken by many US states who have restricted the sale of delta-8-THC until product standards can be developed (Leas 2021).

The finding that a single subreddit provided more AE reports than the FAERS systems highlights the potential of Reddit to serve as a data source that augments voluntary reporting systems such as FAERS and National Poison Control Centers, particularly for novel psychoactive substances. Increases in the number of mentions of novel psychoactive substances on Reddit typically presage increases in the observed number of exposures reported for these substances, facilitating rapid identification of emerging trends (Barenholtz et al. 2021). The grouping of Reddit into subreddits–with more than 600 related to substance use–also help to serve as a filter for identifying posts about a substance even when they do not include the names of products, unlike typical search strategies on other platforms (Adams et al. 2019). Additionally, Reddit allows users unlimited characters which allows them to explain their AEs in more detail than is possible on other platforms such as Twitter.

### Limitations

This study provides a window into adverse events that delta-8-THC users have experienced, but some limitations should be noted. While our analyses demonstrate concordance with the AEs reported on r/Delta8 and FAERS, differences noted for PTs could stem from differences in the coding strategy between our team and the FDA’s and not difference in case presentation. Additionally, the extent to which either of these data sources can be generalized to the larger population remains unknown, because no external comparisons exist. In general, Reddit users tend to be younger and male, (Pew Research Center n.d.) but this may vary among subreddits and could not be discerned from the AE reports we studied. The data rely on self-report of delta-8-THC exposure and outcomes which could not be biochemically verified or clinical diagnosed. Future research could explore the extent to which a causal link could be drawn between delta-8-THC and AE reports posted on Reddit and whether Reddit users are responsive to additional direct messaging to queries that might be needed for determining causality. Finally, delta-8-THC is just one of many emerging unregulated cannabinoids (e.g., hexahydrocannabinol or HHC) and future research should explore potential AEs experienced by users of these products as they may differ from delta-8-THC.

## Conclusions

The findings of this case series suggest that the public is experiencing AEs related to delta-8-THC use that typically resemble AEs experienced during acute cannabis intoxication. As such, clinicians may be able to treat and manage most delta-8-THC cases as they would typically manage a case of acute cannabis intoxication. However, cases where delta-8-THC exposure is suspected should also be monitored for symptoms that could result from product contamination. Given the similarity in AEs between delta-8-THC and delta-9-THC, jurisdictions should also consider clarifying whether delta-8-THC can be sold as a hemp product.

## Supplementary Information

**Additional file 1: APPENDIX.** Self-reported AdverseEvents Associated with ∆^8^-Tetrahydrocannabinol (Delta-8-THC) Use. **Tablee1.** Serious adverse events associatedwith delta-8-THC reported by delta-8-THC users on Reddit delta-8-THC forum r/Delta8.

## Data Availability

The data this study sampled is publicly available. Access to our coded data can be provided with a reasonable request made to the corresponding author.

## Abbreviations

AE: Adverse Event
r/Delta8: A topically-focused Reddit forum known as a “subreddit” dedicated to discussions of delta-8-THC
CT scan: Computerized tomography scan
Delta-8-THC: ∆^8^*-*Tetrahydrocannabinol
Delta-9-THC: ∆^9^-Tetrahydrocannabinol
ER: Emergency Room
EVALI: E-cigarette or vaping use-associated lung injury
FAERS: The US Food and Drug Administration Adverse Event Reporting System
FDA: U.S. Food and Drug Administration
HHC: Hexahydrocannabinol
MedDRA: Medical Dictionary for Regulatory Activities
PT: MedDRA Preferred Term
SOC: MedDRA System Organ Class
LLT: MedDRA Lowest-Level Term
