# Supplementary material for: Self-reported adverse events associated with ∆8-Tetrahydrocannabinol (Delta-8-THC) Use
Source: J Cannabis Res. 2023 May 23;5:15. doi: 10.1186/s42238-023-00191-y (PMC10204335; doi:10.1186/s42238-023-00191-y)
Supplement: Supplementary file 1 — Additional file 1: APPENDIX. Self-reported AdverseEvents Associated with ∆8-Tetrahydrocannabinol (Delta-8-THC) Use. Tablee1. Serious adverse events associatedwith delta-8-THC reported by delta-8-THC users on Reddit delta-8-THC forum r/Delta8. [file 42238_2023_191_MOESM1_ESM.docx]

**APPENDIX**

**Self-reported Adverse Events Associated with ∆^8^-Tetrahydrocannabinol (Delta-8-THC) Use**

Table e1. Serious adverse events associated with delta-8-THC reported by delta-8-THC users on Reddit delta-8-THC forum r/Delta8……………….….….……….….….….….….….……Page 2

**Table e1. Serious adverse events associated with delta-8-THC reported by delta-8-THC users on Reddit delta-8-THC forum (r/Delta8)**

| **Serious AE Case Number** | **Preferred Terms Associated with Serious AE** | **System Organ Class of Preferred Terms** | **Outcome** |
| --- | --- | --- | --- |
| 1 | Chest discomfort | General disorders and administration site conditions | Other Outcomes |
|  | Head discomfort* | Nervous system disorders |  |
| 2 | Decreased appetite | Metabolism and nutrition disorders | Other Outcomes |
|  | Withdrawal syndrome | General disorders and administration site conditions |  |
| 3 | Depression | Psychiatric disorders | Required Intervention |
|  | Emotional poverty* | Psychiatric disorders |  |
| 4 | Dyspnoea | Respiratory, thoracic and mediastinal disorders | Other Outcomes |
|  | Rhinorrhoea* | Respiratory, thoracic and mediastinal disorders |  |
| 5 | Hypertension | Vascular disorders | Hospitalized |
| 6 | Hypersensitivity | Immune system disorders | Other Outcomes |
|  | Odynophagia* | Gastrointestinal disorders |  |
|  | Pharyngeal swelling* | Respiratory, thoracic and mediastinal disorders |  |
| 7 | Drug withdrawal syndrome | General disorders and administration site conditions | Other Outcomes |
|  | Nausea | Gastrointestinal disorders |  |
|  | Vomiting | Gastrointestinal disorders |  |
| 8 | Hypertension | Vascular disorders | Hospitalized |
|  | Vision blurred | Eye disorders |  |
|  | Seizure | Nervous system disorders |  |
|  | Dizziness | Nervous system disorders |  |
|  | Pyrexia | General disorders and administration site conditions |  |
|  | Heart rate increased | Investigations |  |
|  | Shock | Vascular disorders |  |
|  | Vertigo | Ear and labyrinth disorders |  |
|  | Hallucination, visual | Psychiatric disorders |  |
|  | Vomiting | Gastrointestinal disorders |  |
| 9 | Chills | General disorders and administration site conditions | Other Outcomes |
|  | Insomnia | Psychiatric disorders |  |
| 10 | Feeling abnormal | General disorders and administration site conditions | Required Intervention |
| 11 | Hypoaesthesia | Nervous system disorders | Other Outcomes |
|  | Seizure | Nervous system disorders |  |
|  | Paranoia | Psychiatric disorders |  |
|  | Vomiting | Gastrointestinal disorders |  |
| 12 | Sensory disturbance | Nervous system disorders | Other Outcomes |
|  | Chills | General disorders and administration site conditions |  |
|  | Dyspnoea | Respiratory, thoracic and mediastinal disorders |  |
|  | Dizziness | Nervous system disorders |  |
|  | Vomiting | Gastrointestinal disorders |  |
| 13 | Chest pain | General disorders and administration site conditions | Other Outcomes |
|  | Feeling abnormal | General disorders and administration site conditions |  |
|  | Amnesia | Nervous system disorders |  |
|  | Neuralgia* | Nervous system disorders |  |
|  | Libido increased* | Psychiatric disorders |  |
|  | Abdominal pain upper | Gastrointestinal disorders |  |
|  | Axillary pain* | General disorders and administration site conditions |  |
| 14 | Dyspnoea | Respiratory, thoracic and mediastinal disorders | Other Outcomes |
|  | Paranoia | Psychiatric disorders |  |
| 15 | Hallucinations, mixed | Psychiatric disorders | Other Outcomes |
|  | Paranoia | Psychiatric disorders |  |
|  | Hallucination, visual | Psychiatric disorders |  |
|  | Metamorphopsia* | Eye disorders |  |
| 16 | Drug withdrawal syndrome | General disorders and administration site conditions | Other Outcomes |
|  | Fatigue | General disorders and administration site conditions |  |
|  | Headache | Nervous system disorders |  |
|  | Laziness* | Psychiatric disorders |  |
| 17 | Anxiety | Psychiatric disorders | Other Outcomes |
|  | Depressed mood* | Psychiatric disorders |  |
|  | Withdrawal syndrome | General disorders and administration site conditions |  |
| 18 | Fatigue | General disorders and administration site conditions | Other Outcomes |
|  | Withdrawal syndrome | General disorders and administration site conditions |  |
| 19 | Anxiety | Psychiatric disorders | Other Outcomes |
|  | Depression | Psychiatric disorders |  |
|  | Drug withdrawal syndrome | General disorders and administration site conditions |  |
|  | Insomnia | Psychiatric disorders |  |
|  | Neurological symptom* | Nervous system disorders |  |
|  | Appetite disorder* | Metabolism and nutrition disorders |  |
| 20 | Chills | General disorders and administration site conditions | Other Outcomes |
|  | Dyspnoea | Respiratory, thoracic and mediastinal disorders |  |
|  | Dizziness | Nervous system disorders |  |
|  | Blood pressure increased | Investigations |  |
|  | Heart rate increased | Investigations |  |
|  | Amnesia | Nervous system disorders |  |
|  | Muscle spasms | Musculoskeletal and connective tissue disorders |  |
|  | Paranoia | Psychiatric disorders |  |
|  | Tremor | Nervous system disorders |  |
|  | Swollen tongue | Gastrointestinal disorders |  |
|  | Vomiting | Gastrointestinal disorders |  |
| 21 | Chest discomfort | General disorders and administration site conditions | Other Outcomes |
|  | Dyspnoea | Respiratory, thoracic and mediastinal disorders |  |
| 22 | Haemoptysis* | Respiratory, thoracic and mediastinal disorders | Other Outcomes |
| 23 | Panic attack | Psychiatric disorders | Hospitalized |
|  | Dysphemia* | Psychiatric disorders |  |
|  | Muscle twitching | Musculoskeletal and connective tissue disorders |  |
| 24 | Dyspnoea | Respiratory, thoracic and mediastinal disorders | Other Outcomes |
| 25 | Abdominal pain | Gastrointestinal disorders | Other Outcomes |
|  | Intra-abdominal fluid collection* | Gastrointestinal disorders |  |
| 26 | Chest discomfort | General disorders and administration site conditions | Hospitalized, Other Outcomes |
|  | Dizziness | Nervous system disorders |  |
|  | Dry eye* | Eye disorders |  |
|  | Dry mouth | Gastrointestinal disorders |  |
|  | Fatigue | General disorders and administration site conditions |  |
|  | Eye pain* | Eye disorders |  |
|  | Headache | Nervous system disorders |  |
|  | Heart rate increased | Investigations |  |
|  | Muscle spasms | Musculoskeletal and connective tissue disorders |  |
|  | Dysarthria | Nervous system disorders |  |
|  | Muscle twitching | Musculoskeletal and connective tissue disorders |  |
|  | Vomiting | Gastrointestinal disorders |  |
|  | Blepharospasm* | Eye disorders |  |
|  | Metamorphopsia* | Eye disorders |  |
|  | Balance disorder | Nervous system disorders |  |
| 27 | Accidental overdose | Injury, poisoning and procedural complications | Hospitalized |
|  | Panic reaction | Psychiatric disorders |  |
| 28 | Dyspnoea | Respiratory, thoracic and mediastinal disorders | Other Outcomes |
|  | Panic attack | Psychiatric disorders |  |
|  | Paranoia | Psychiatric disorders |  |
| 29 | Loss of consciousness | Nervous system disorders | Other Outcomes |
|  | Cough | Respiratory, thoracic and mediastinal disorders |  |
|  | Dizziness | Nervous system disorders |  |
|  | Panic attack | Psychiatric disorders |  |
| 30 | Dependence* | Psychiatric disorders | Other Outcomes |
| 31 | Chest pain | General disorders and administration site conditions | Required Intervention, Other Outcomes |
|  | Productive cough* | Respiratory, thoracic and mediastinal disorders |  |
|  | Secretion discharge* | General disorders and administration site conditions |  |
| 32 | Chest pain | General disorders and administration site conditions | Other Outcomes |
|  | Dyspnoea | Respiratory, thoracic and mediastinal disorders |  |
| 33 | Anxiety | Psychiatric disorders | Other Outcomes |
|  | Decreased appetite | Metabolism and nutrition disorders |  |
|  | Insomnia | Psychiatric disorders |  |
|  | Night sweats | Skin and subcutaneous tissue disorders |  |
|  | Withdrawal syndrome | General disorders and administration site conditions |  |
| 34 | Cough | Respiratory, thoracic and mediastinal disorders | Other Outcomes |
| 35 | Cough | Respiratory, thoracic and mediastinal disorders | Other Outcomes |
|  | Dyspnoea | Respiratory, thoracic and mediastinal disorders |  |
| 36 | Chest discomfort | General disorders and administration site conditions | Other Outcomes |
|  | Feeling hot | General disorders and administration site conditions |  |
|  | Unevaluable event | General disorders and administration site conditions |  |
| 37 | Loss of consciousness | Nervous system disorders | Life Threatening, Required Intervention, Other Outcomes |
|  | Vision blurred | Eye disorders |  |
|  | Chills | General disorders and administration site conditions |  |
|  | Seizure | Nervous system disorders |  |
|  | Heart rate increased | Investigations |  |
|  | Tremor | Nervous system disorders |  |
|  | Dysarthria | Nervous system disorders |  |
| 38 | Dependence* | Psychiatric disorders | Other Outcomes |
|  | Unevaluable event | General disorders and administration site conditions |  |
| 39 | Chest pain | General disorders and administration site conditions | Other Outcomes |
|  | Chest discomfort | General disorders and administration site conditions |  |
|  | Cough | Respiratory, thoracic and mediastinal disorders |  |
|  | Productive cough* | Respiratory, thoracic and mediastinal disorders |  |
| 40 | Anxiety | Psychiatric disorders | Required Intervention |
|  | Cough | Respiratory, thoracic and mediastinal disorders |  |
|  | Dizziness | Nervous system disorders |  |
|  | Heart rate increased | Investigations |  |
|  | Urinary retention* | Renal and urinary disorders |  |
| 41 | Dependence* | Psychiatric disorders | Other Outcomes |
|  | Headache | Nervous system disorders |  |
|  | Vomiting | Gastrointestinal disorders |  |
| 42 | Chest discomfort | General disorders and administration site conditions | Required Intervention, Other Outcomes |
|  | Vascular pain* | Vascular disorders |  |
|  | Rash | Skin and subcutaneous tissue disorders |  |
| 43 | Dependence* | Psychiatric disorders | Other Outcomes |
|  | Irritability | Psychiatric disorders |  |
| 44 | Chest pain | General disorders and administration site conditions | Other Outcomes |
| 45 | Anxiety | Psychiatric disorders | Other Outcomes |
|  | Disturbance in attention | Nervous system disorders |  |
|  | Heart rate increased | Investigations |  |
|  | Irritability | Psychiatric disorders |  |
|  | Withdrawal syndrome | General disorders and administration site conditions |  |
| 46 | Abnormal dreams | Psychiatric disorders | Other Outcomes |
|  | Dizziness | Nervous system disorders |  |
|  | Withdrawal syndrome | General disorders and administration site conditions |  |
| 47 | Anxiety | Psychiatric disorders | Other Outcomes |
|  | Depression | Psychiatric disorders |  |
|  | Apathy | Psychiatric disorders |  |
|  | Panic attack | Psychiatric disorders |  |
| 48 | Dyspnoea | Respiratory, thoracic and mediastinal disorders | Other Outcomes |
|  | Nausea | Gastrointestinal disorders |  |
| 49 | Ammonia increased* | Investigations | Hospitalized |
|  | Hepatic encephalopathy* | Nervous system disorders |  |
|  | Ill-defined disorder* | General disorders and administration site conditions |  |
| 50 | Dependence* | Psychiatric disorders | Other Outcomes |
|  | Anxiety | Psychiatric disorders |  |
|  | Amnesia | Nervous system disorders |  |
|  | Panic attack | Psychiatric disorders |  |
| 51 | Chest discomfort | General disorders and administration site conditions | Other Outcomes |
|  | Dyspnoea | Respiratory, thoracic and mediastinal disorders |  |
|  | Presyncope | Nervous system disorders |  |
| 52 | Nausea | Gastrointestinal disorders | Other Outcomes |
|  | Withdrawal syndrome | General disorders and administration site conditions |  |
| 53 | Chest discomfort | General disorders and administration site conditions | Other Outcomes |
|  | Dyspnoea | Respiratory, thoracic and mediastinal disorders |  |
| 54 | Chest discomfort | General disorders and administration site conditions | Other Outcomes |
|  | Pulmonary pain* | Respiratory, thoracic and mediastinal disorders |  |
| 55 | Anxiety | Psychiatric disorders | Required Intervention |
|  | Chest pain | General disorders and administration site conditions |  |
| 56 | Chest discomfort | General disorders and administration site conditions | Required Intervention |
|  | Asthma* | Respiratory, thoracic and mediastinal disorders |  |
|  | Productive cough* | Respiratory, thoracic and mediastinal disorders |  |
|  | Throat tightness | Respiratory, thoracic and mediastinal disorders |  |
| 57 | Abdominal pain | Gastrointestinal disorders | Other Outcomes |
|  | Abnormal dreams | Psychiatric disorders |  |
|  | Anxiety | Psychiatric disorders |  |
|  | Hypertension | Vascular disorders |  |
|  | Diarrhoea | Gastrointestinal disorders |  |
|  | Dyspnoea | Respiratory, thoracic and mediastinal disorders |  |
|  | Gastrooesophageal reflux disease | Gastrointestinal disorders |  |
| 58 | Choking sensation | Respiratory, thoracic and mediastinal disorders | Required Intervention, Other Outcomes |
|  | Dyspnoea | Respiratory, thoracic and mediastinal disorders |  |
|  | Hepatomegaly* | Hepatobiliary disorders |  |
|  | Panic attack | Psychiatric disorders |  |
|  | Tremor | Nervous system disorders |  |
|  | Vomiting | Gastrointestinal disorders |  |
|  | Ill-defined disorder* | General disorders and administration site conditions |  |
|  | Unevaluable event | General disorders and administration site conditions |  |
| 59 | Dizziness | Nervous system disorders | Required Intervention |
|  | Heart rate increased | Investigations |  |
|  | Nausea | Gastrointestinal disorders |  |
|  | Palpitations | Cardiac disorders |  |
|  | Tremor | Nervous system disorders |  |
|  | Hyperhidrosis | Skin and subcutaneous tissue disorders |  |
| 60 | Chest pain | General disorders and administration site conditions | Other Outcomes |
|  | Oropharyngeal pain | Respiratory, thoracic and mediastinal disorders |  |
| 61 | Withdrawal syndrome | General disorders and administration site conditions | Other Outcomes |
| 62 | Abnormal dreams | Psychiatric disorders | Other Outcomes |
|  | Decreased appetite | Metabolism and nutrition disorders |  |
|  | Disturbance in attention | Nervous system disorders |  |
|  | Chills | General disorders and administration site conditions |  |
|  | Depression | Psychiatric disorders |  |
|  | Insomnia | Psychiatric disorders |  |
|  | Nervousness | Psychiatric disorders |  |
|  | Hyperhidrosis | Skin and subcutaneous tissue disorders |  |
|  | Thirst decreased* | General disorders and administration site conditions |  |
|  | Withdrawal syndrome | General disorders and administration site conditions |  |
| 63 | Depression | Psychiatric disorders | Other Outcomes |
|  | Drug withdrawal syndrome | General disorders and administration site conditions |  |
|  | Nausea | Gastrointestinal disorders |  |
|  | Vomiting | Gastrointestinal disorders |  |
| 64 | Chest pain | General disorders and administration site conditions | Required Intervention, Other Outcomes |
|  | Chills | General disorders and administration site conditions |  |
|  | Dyspnoea | Respiratory, thoracic and mediastinal disorders |  |
|  | Dizziness | Nervous system disorders |  |
|  | Heart rate increased | Investigations |  |
|  | Hunger | General disorders and administration site conditions |  |
|  | Sedation | Nervous system disorders |  |
|  | Energy increased* | General disorders and administration site conditions |  |
|  | Negative thoughts | Psychiatric disorders |  |
| 65 | Perihepatic discomfort* | Hepatobiliary disorders | Other Outcomes |
|  | Unevaluable event | General disorders and administration site conditions |  |
| 66 | Chest pain | General disorders and administration site conditions | Other Outcomes |
|  | Dyspnoea | Respiratory, thoracic and mediastinal disorders |  |
|  | Oropharyngeal pain | Respiratory, thoracic and mediastinal disorders |  |
| 67 | Blood pressure increased | Investigations | Required Intervention |
|  | Heart rate increased | Investigations |  |
|  | Panic attack | Psychiatric disorders |  |
|  | Presyncope | Nervous system disorders |  |

* Indicates a MedDRA preferred term that was not listed in a FAERS case

Abbreviations: FAERS = Food and Drug Administration Adverse Event Reporting System; MedDRA = Medical Dictionary for Regulatory Activities
